# Effects of Boric Acid Gel on Vaginal *Candida albicans* Infections and the Local Immune System in Mice

**DOI:** 10.3389/fimmu.2022.950215

**Published:** 2022-07-25

**Authors:** Xiaoyu Guo, Tingting Jing, Xiaojing Li, Zhao Liu, Yongxue Chen, Yiquan Li, Yanyan Xu, Hongqi Gao

**Affiliations:** ^1^ Department of Clinical Medicine, Hebei University of Engineering, Handan, China; ^2^ Affiliated Hospital of Hebei University of Engineering, Handan, China; ^3^ Department of Dermatology, Affiliated Hospital of Hebei University of Engineering, Handan, China; ^4^ Department of Anesthesiology, Handan Central Hospital, Handan, China; ^5^ Department of Health Services, Logistics University of People’s Armed Police Force, Tianjin, China

**Keywords:** *Candida albicans*, immune system, cytokines, mice, boric acid gel

## Abstract

The objective was to determine the effect of 5% boric acid gel on vaginal *Candida albicans* (CA) infections in mice and its effect on the local immune system (i.e., Th1, Th2, and Th17). Female mice were divided into four groups, with 10 mice in each group. Mycelial suspensions were administered into the vaginal lumen close to the cervix in groups B, F, and M. Mice in group B were given boric acid gel, and group F was treated with fluconazole gel for 30 min every 12 h. Group M was treated with sterile water, and group N was not given treatment. After the seventh day of treatment, each group was observed with the naked eye, and vaginal lavage fluid and vaginal tissue were collected. Expression levels of cytokines were measured using enzyme-linked immunosorbent assays (ELISA) and immunohistochemistry. Periodic acid Schiff (PAS) staining was used to measure the fungi in vaginal tissues. There were no significant changes in group M. In groups B and F, there was less vaginal injury and less exudate, with group B doing better than group F. The numbers of CA colonies were higher in groups B, F, and M than in group N (*P* < 0.01). There was less vaginal colonization of CA in group B than in group F (*P* < 0.01). After the seventh day of treatment, levels of IFN-γ, IL-17, IL-6, TGF-β, IL-4, and IL-10 were significantly greater in groups B, F, and M than in group N (*P* < 0.001); levels of IFN-γ, IL-17, IL-6, and TGF-β in groups B and F were higher than those of group M (*P* < 0.01), while IL-4 and IL-10 levels were significantly lower (*P* < 0.001). The trends of cytokine increases and decreases were more significant in group B than in group F (*P* < 0.05). Immunohistochemical results were similar to ELISA results. PAS staining revealed that boric acid inhibited hyphal reproduction. The boric acid significantly reduced the symptoms associated with CA vaginal infection. It inhibited the CA growth, prevented vaginal lesions, promoted the secretion of Th1 and Th17 cytokines, and reduced Th2 cytokines.

## Introduction

*Candida* is a symbiotic fungus widely present in humans and animals. Usually not pathogenic, *Candida* can cause infections when the fungus overproliferates or the host is immunocompromised. *Candida albicans* (CA) has the highest pathogenicity rate (up to 90%) (1). When the local immune defense ability of the vagina is insufficient, CA is overproduced, and the pathogenicity of the strain is enhanced. The microenvironment in the vagina is very likely to be unbalanced, inducing vulvovaginal candidiasis (VVC).

VVC can occur in more than 70% of the global female population (2). Women of childbearing age are the most at risk. Clinical manifestations include vulvovaginal itching, burning pain, vulvar redness and swelling, increased vaginal discharge, and secretions, accompanied by urinary symptoms and symptoms such as dyspareunia, and can even lead to sexual dysfunction. Due to improper treatment, recurrent or prolonged illness may lead to recurrent vulvovaginal candidiasis (RVVC) (3). The incidence of VVC is increasing, and some individuals are susceptible to recurrent episodes. For these reasons, cost-effective antifungal agents have become a top priority for the treatment of mycosis.

Investigators have proposed that the adaptive immune response is associated with the pathogenesis and treatment of VVC; *Candida* overload in the vagina induces neutrophil aggregation, triggering innate immunity designed to clear the organism while manifesting infection symptoms and generating treatment signals (4, 5). In addition to mediating local immune responses in mucous membranes, neutrophils significantly enhance the uptake of hyphal strains by innate immune cells (e.g., macrophages and dendritic cells), induce the secretion of cytokines (i.e., IL-12 and IL-4), and regulate the differentiation of CD4+ T lymphocytes into helper T lymphocytes (Th) and regulatory T lymphocytes (Tregs) (4). The former differentiate into Th1, Th2, and Th17. By secreting IFN-γ, IL-4, IL-6, and TGF-β, et al. (6–8), the adaptive immune response is induced to participate in pathogenesis (9, 10).

There is a lack of research on activating antifungal properties by influencing the immune responses. Boric acid has been used for decades as a safe, effective, low-resistance fungal bacteriostatic agent for VVC treatment (11, 12). Khameneie et al. conducted clinical studies on fluconazole and boric acid’s efficacy in treating VVC and found that boric acid replaced azole drugs and was effective against CA and *Candida glabrases (* 13). Guaschino et al. found no significant difference in the clinical efficacy of boric acid and itraconazole in RVVC treatment (14). Previous studies by Cenci E et al. demonstrated the effect of fluconazole on Th1-type reaction, which increased the secretion of IFN-γ and IL-12 while inhibiting the production of IL-4 (15). However, there no study has been found on the effect of boric acid on host local immune function.

In this study, a mouse VVC model was constructed, and the effect of 5% boric acid gel treatment on the local adaptive immune response of the host was explored by monitoring locally adaptive cytokines, and was investigated to provide an experimental basis for the selection of clinically valuable medications.

## Materials and Methods

### Laboratory Animals and Strains

Healthy female ICR (Institute of Cancer Research) mice aged 6–8 weeks and weighing 20–25 g were purchased from Beijing Weitong Lihua Experimental Animal Technology Co., LTD. The ATCC 66027 C*.albicians* was purchased from the American Type Culture Collection.

### Reagents and Instruments

Boric acid 5% gel was provided by the Laboratory of Fungal Molecular Biology, Shanghai Changzheng Hospital. The ELISA kits were purchased from Wuhan Huamei Company (China). The primary and secondary antibodies of Immunohistochemistry were provided by Bioss Co., LTD (China), Abcam Co. (UK), and Beijing Zhongshan Golden Bridge Co. (China).

### Preparation of CA Liquid

The standard strain of CA (ATCC 66027) stored at –4°C was thawed at room temperature. The concentration of fungi was adjusted to 1.0 × 10^8^ CFU/ml through purification and excitation.

### Preparation of Fluconazole Gel

Fluconazole gel preparation was based on a published method (16). The specific steps were as follows. Preparation of A: we evenly spread 9 g of carbomer on the surface of 300 g purified water and let it stand for 24 h until fully swollen. Preparation of B: we added 15 g of fluconazole fine powder to a mixture of 190 g ethanol and 100 g phosphate buffer (pH 7.8) after passing and then 300 g of purified water to mix and stir until dissolved. Finally, we added B to A, stirred and mixed well, added 19 g triethanolamine, stirred while adding, added purified water until to 1000 g, and stirred well.

### Model Construction and Group Intervention

Mice were randomly divided into four groups of ten mice each. The mouse model of vaginal infection was generated as described elsewhere (17). Three days before infection, pseudoestrus was induced by subcutaneous injection of 0.1 ml estradiol benzoate oil agent (1 mg/mL) (Henan Zhongnongkang Livestock Trade Co. LTD). The injection was administered every other day throughout the experiment. Mice were inoculated with 20 μL of 1.0 × 10^8^ CFU/ml mycelial suspensions and administered using a mechanical pipette into the vaginal lumen close to the cervix in the M, F, and B groups. The N group was treated with sterile water. After the third day of inoculation, we determined whether the mouse vagina CA infection model was successfully generated using fungal fluorescence microscopy and colony counts.

After the model was successfully generated, each mouse in group B was given 5% boric acid gel. Group F was treated with fluconazole gel for 30 min every 12 h. Group M was treated with sterile water, while group N was not treated for seven days.

### Sample Collection

a: Macroscopic images were taken before and the seventh day after administration.

b: After the seventh day, colonies were observed and counted.

c: Supernatants from vaginal lavage fluid (1 ml) were collected before and seven days after administration. IFN-γ, IL-17, IL-6, TGF-β, IL-4, IL-10 levels were measured using ELISA kits.

d: After the seventh day of treatment, the mice were sacrificed using deep anesthesia, and the vaginal tissue was removed and immediately fixed in 4% neutral buffered formalin for 24 h. Specimens were then dehydrated and paraffin-embedded. PAS staining was used to observe the changes in the number of mycelium adhesions before and after treatment. Expression of IFN-γ, IL-17, IL-6, TGF-β, IL-4, and IL-10 was observed using immunohistochemistry before and after treatment.

### Fluorescence Microscopy

On the third day after inoculation, three mice were randomly selected from each group (a total of twelve mice). Vaginal lavage solution (10 μL) was mixed with sterile phosphate-buffered saline, then dropped on the glass slide, and added fluorescence staining solution for fungal detection. Coverslips were used for observation under a microscope.

### Colony Counts

Three mice were randomly selected from each group (12 mice). We took 20 μL of sterile phosphate-buffered saline vaginal lavage solution and diluted, centrifuged, and removed the supernatants. We then inoculated them into Sabouraud Dextrose Agar (SDA) medium, incubated them at 35°C for 48 h, and counted colonies.

### ELISA

We used microplates coated with IFN-γ-specific antibody, the standard product, sample (mouse vaginal lavage solution), horseradish peroxidase-specific antibody, catalytic enzyme, and color developer added sequentially, and the absorbance (optical density). The reaction product was measured using a microplate at the absorbance at 450 nm to determine concentration. The determination principle and procedures of IL-6, IL-17, TGF-β, IL-10, and IL-4 content in vaginal lavage fluid was consistent with that of IFN-γ.

### Periodic Acid-Schiff Staining

For histological analysis, the mice were sacrificed, and vaginal tissue was removed and immediately fixed in 4% neutral buffered formalin for 24 h. The vaginal specimens were then dehydrated, paraffin-embedded, and 3–4 μm sections were prepared. The sections were stained with PAS.

### Immunohistochemistry

Sections were soaked in xylene three times for 15 min each, followed by anhydrous ethanol twice for five minutes each. The residue was washed off and sections soaked in 3% hydrogen peroxide solution for 25 minutes, then decolorized three times for 5 minutes each. We added 3% bovine serum albumin (BSA) dropwise to cover the tissue, after 30 min. and successively added goat serum and rabbit serum overnight. decolorized three times. We added IFN-γ secondary antibody, after 50 min, decolorized three times again. We dropwise added DAB color developer, followed for drying and sealing. The digital microscope was used for observation and analysis. IL-6, IL-17, TGF-β, IL-10, and IL-4 were determined using the same detection step as IFN-γ.

### Statistical Analysis

SPSS25.0 software was used for statistical analysis. To determine statistical significance, we used unpaired t-tests (with or without Welch’s correction, as appropriate), one-way analysis of variance with the lease square distance t-test, or Kruskal-Wallis test with Dunnett’s *post hoc* multiple-comparison test. Quantitative data were expressed as means ± standard error of the mean. The grade data were compared using the Kruskal-Wallis rank test between multiple independent groups of independent samples, and the Mann-Whitney test was used for two independent samples. *P*-values of < 0.05 were considered significant.

## Results

### Clinical Manifestations

**Figure 1:**
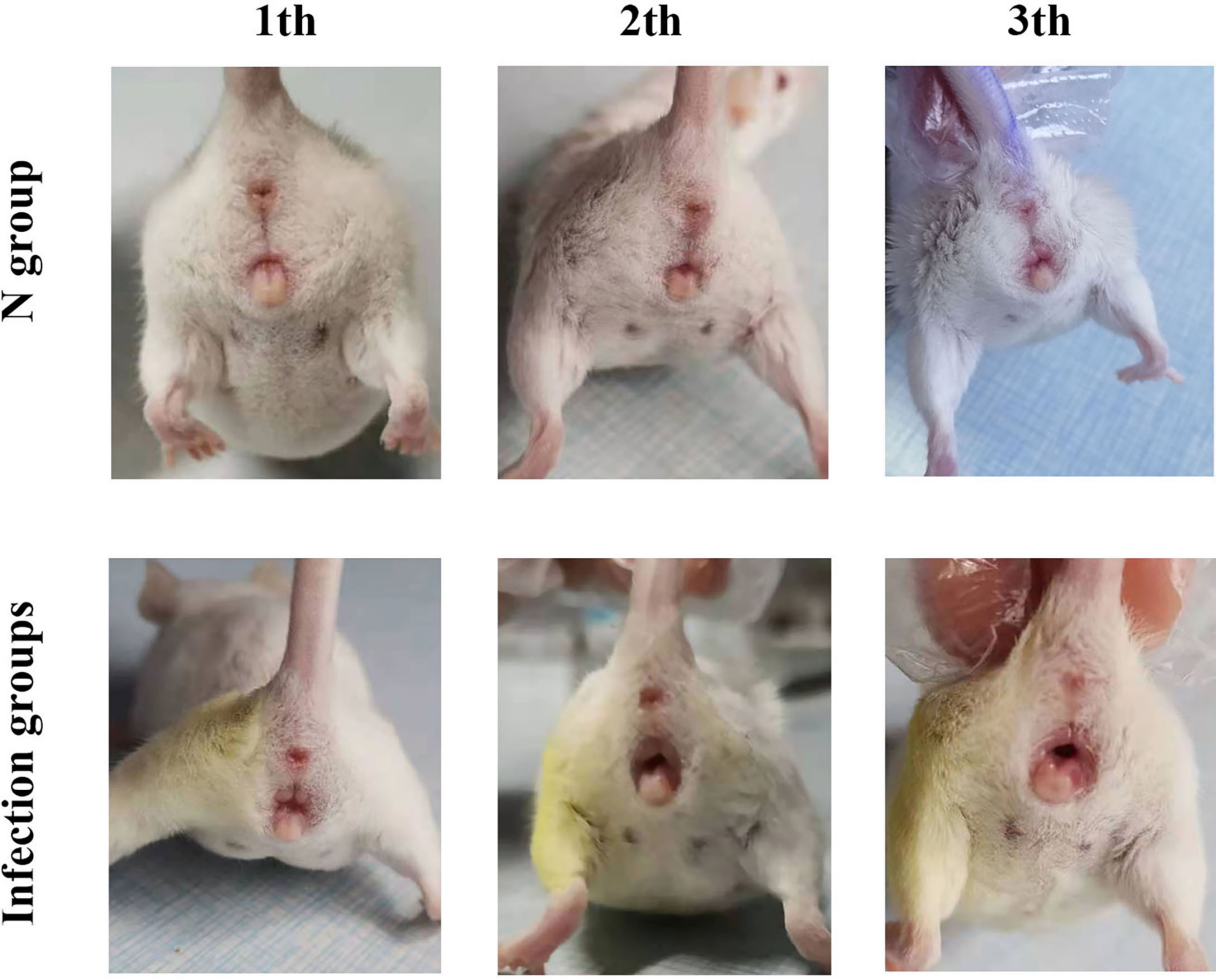
Clinical manifestations. On the third day after inoculation in the three infection groups, there was vulvar redness and erosion, and the mice engaged in continuous licking behaviors.

After inoculating CA ATCC 66027 (**Figure 1**), the vaginal model was generated in the M, F, and B groups. Redness, swelling, exudation increase, erosions, and ulcers could be seen. There were no changes in group N.

### Fluorescence Microscopy and Colony Counts

**Figure 2:**
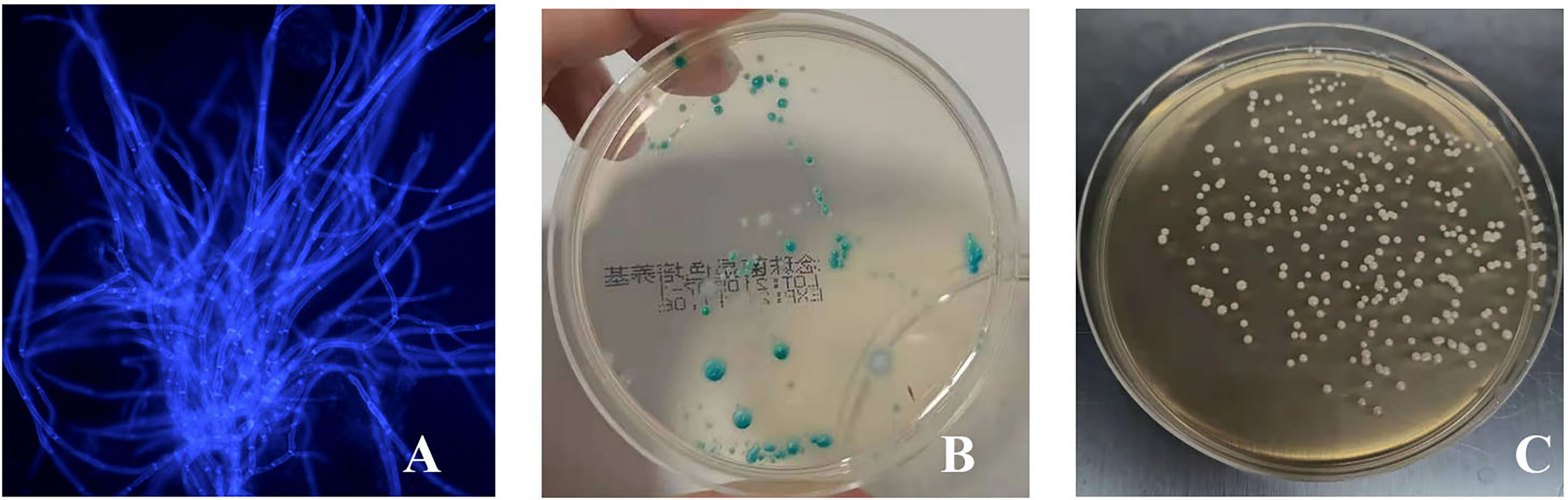
The vaginal infection model was successfully established. **(A)** Lump hyphae were visible by fluorescence microscopic examination (400 ×); **(B)** Culture of chromogenic screening medium; **(C)** The amount of fungal burden in the vagina was higher in the infected mice: colony counts were 1.5 × 10^5^–10^6^ CFU/ml.

On the third day after inoculation of CA ATCC 66027 (**Figure 2A**), there were agglomerated hyphae in groups M, F, and B. Group N was negative.

CA was cultured in a chromogenic screening medium on the third day after inoculation of CA ATCC 66027. CA appeared emerald-green with smooth colonies in groups M, F, and B (**Figure 2B**). CA was negative in group N. Microscopic observation, and counting showed that M and B groups had higher fungal loads, about 1.5 × 10^5^–10^6^ CFU/ml (**Figure 2C**).

### Therapeutic Effect

**Figure 3:**
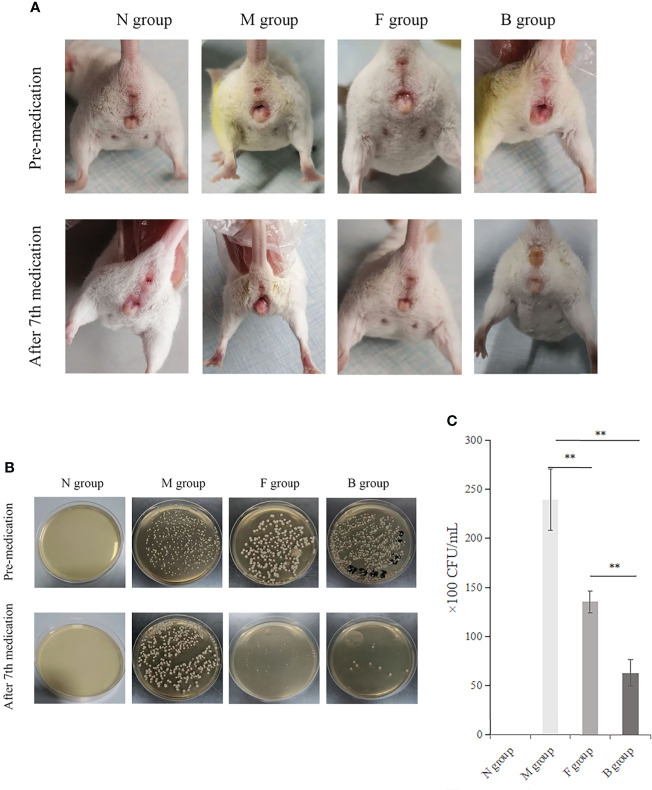
Therapeutic effect. **(A)** Comparison of four groups before and after the seventh day of treatment. **(B)** Comparison of fungal culture before and after medication: Group B had the best therapeutic effect, followed by group F. **(C)** Comparison of bacterial colony count in vaginal lavage fluid of mice after the seventh day of administration, ^**^ *P* < 0.01.

There was no irritation after treatment, and the skin lesions at the modeling site were observed with the naked eye on the seventh day (**Figure 3A**). There was no apparent change in group N; however, erosions could be seen in group M, which was not significantly better than before treatment. In group B, there was less injury, suggesting that the wound underwent apparent healing without exudate.

The numbers of CA colonies in the fungal model groups were significantly higher than in group N (*P* < 0.01). CA colonization was significantly lower in group B than in group M, and the number of bacterial colonies cultured in the irrigation solution was significantly lower (*P* < 0.01). There was less vaginal colonization of CA and fewer colony numbers in lavage solution culture in group F than in group M (*P* < 0.01). The colony count reduction in group B was more significant than in group F (**Figures 3B, C**).

### Expressions of Anti-Candidal Th-Dependent Cytokines

#### ELISA

**Figure 4:**
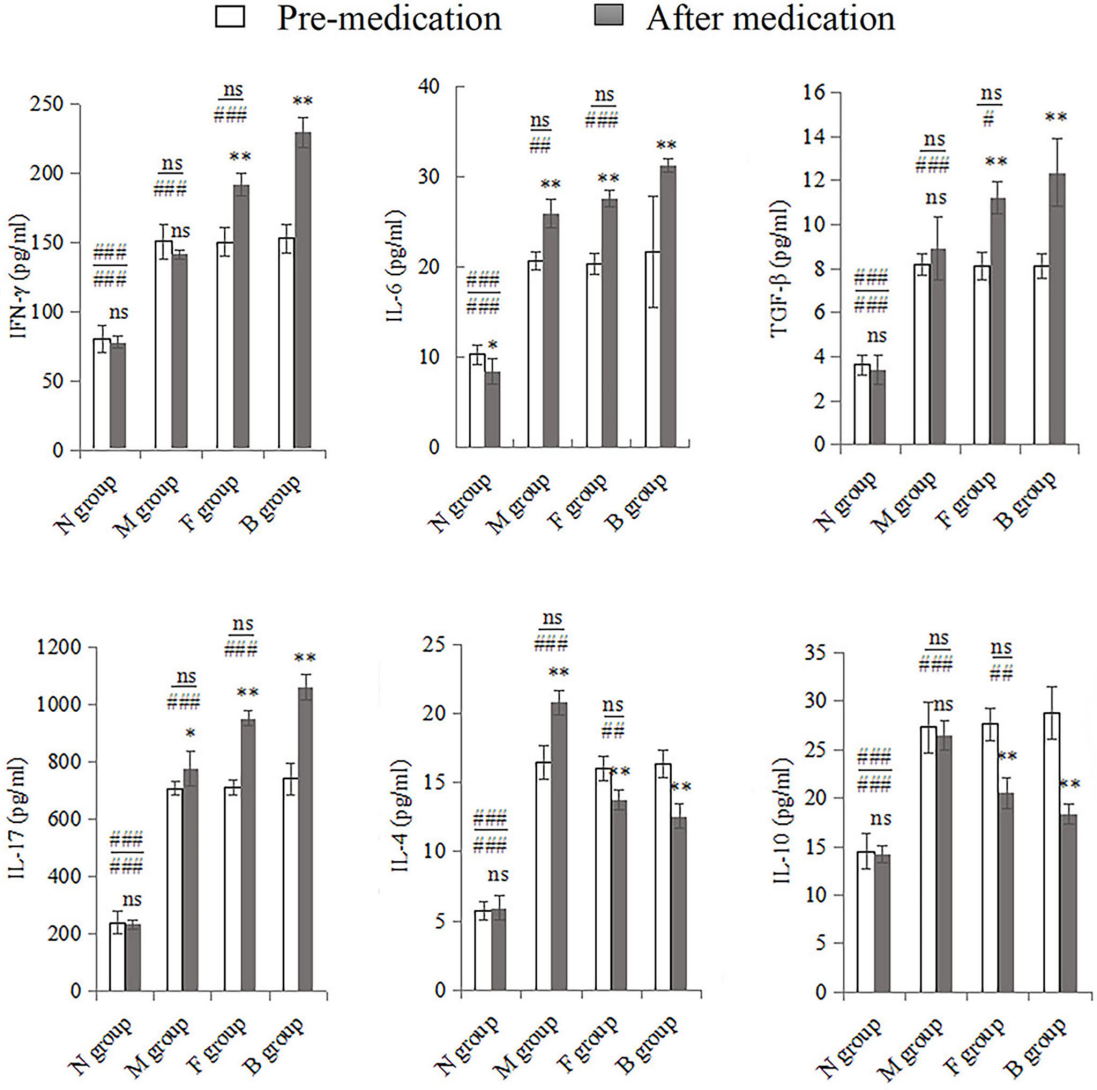
Whole vaginal lavage fluid was subjected to ELISA to measure levels of IFN-γ, IL-17, IL-6, TGF-β, IL-4, and IL-10. All quantitative data were expressed as means ± standard errors of the mean. Comparison of pre-medication and after in each group: ns, not significant, ^*^ *P <* 0.01, ^**^ *P* < 0.001. Above the horizontal line is the comparison before medication between the group and the other three groups, below the line is the comparison after medication between the group and the other three groups: ns: not significant, ^#^ *P <* 0.05, ^##^ *P <* 0.01, ^###^ *P* < 0.001.

There were significant changes in cytokine expression in the CA model groups. After the seventh day of treatment, the mucosal immune factors underwent significant changes compared with pre-medication levels. IL-17, IL-6, IL-4, and TGF-β were higher in group M after treatment than before; only the first three cytokine levels were significantly different (*P* < 0.01), and the expression of IFN-γ and IL-10 decreased slightly (*P* > 0.05). Expression levels of IFN-γ, IL-17, IL-6, and TGF-β in groups F and B were significantly higher after medication than before (*P* < 0.001). IL-4 and IL-10 levels decreased after drug intervention (*P* < 0.001). Before and after treatment, the factors in the three model groups were significantly higher than those in the N group (*P* < 0.001). Pre-medication, there was no significant difference between group M and the other two drug intervention groups (*P* > 0.05); however, IFN-γ, IL-17, IL-6, and TGF-β in groups B and F were higher than in group M after medication (*P* < 0.01), while IL-4 and IL-10 levels were significantly lower (*P* < 0.001). There were no significant differences between groups F and B before medication; however, after medication, IFN-γ, IL-17, IL-6, and TGF-β levels in group B were higher than in group F (*P* < 0.05), and IL-4 and IL-10 levels were lower than in group F (*P* < 0.01; **Figure 4**).

#### Immunohistochemistry

**Figure 5:**
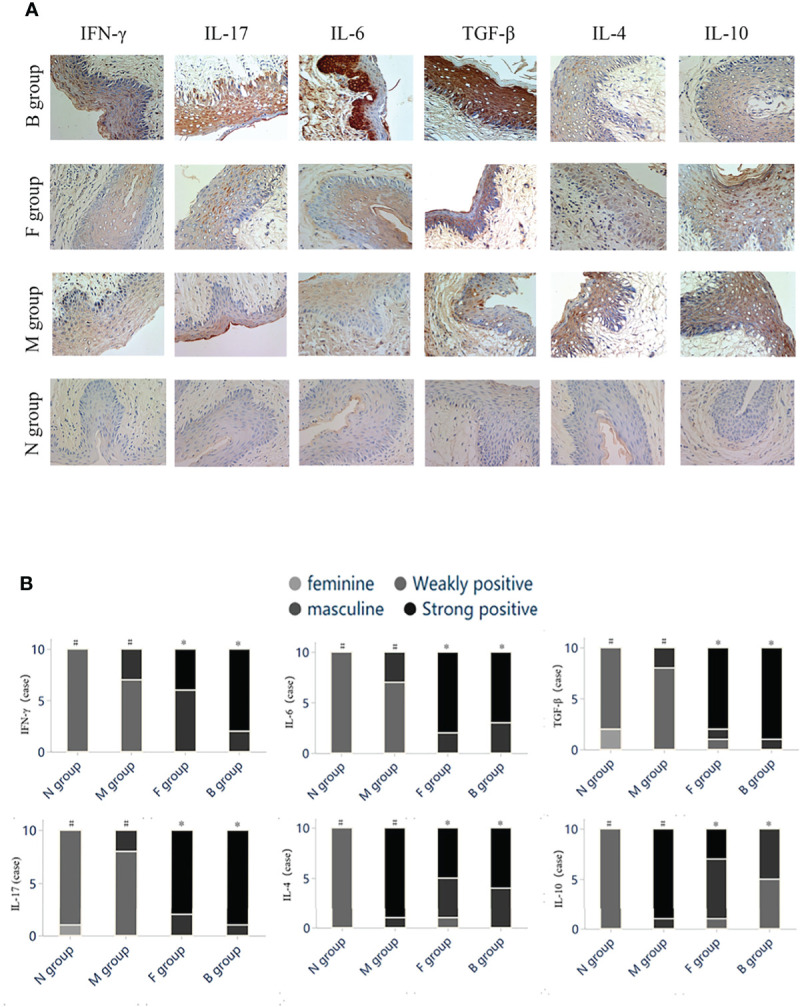
Immunohistochemistry. **(A)** Histochemical staining of cytokines in vaginal tissues of mice in each group. **(B)** The color intensity grading of positive cells was used as the judging basis. The grade data were compared using the Kruskal-Wallis rank test between several groups of independent samples, and the Mann-Whitney test was used for two independent samples. Statistically significant differences are indicated by letters in groups (^#,*^).

After the seventh day of administration, protein expression levels of IFN-γ, IL-17, IL-6, and TGF-β in vaginal tissues were significantly higher in group N than in group M (*P* < 0.05). Expression levels of IFN-γ, IL-17, IL-6, and TGF-β in groups B and F were significantly higher than in group M (*P* < 0.05). Expression levels of anti-inflammatory cytokines IL-4 and IL-10 were significantly lower after drug intervention in groups B, F, and M (*P* < 0.05) (**Figure 5**).

### Fungal Content in Vaginal Tissue

**Figure 6:**
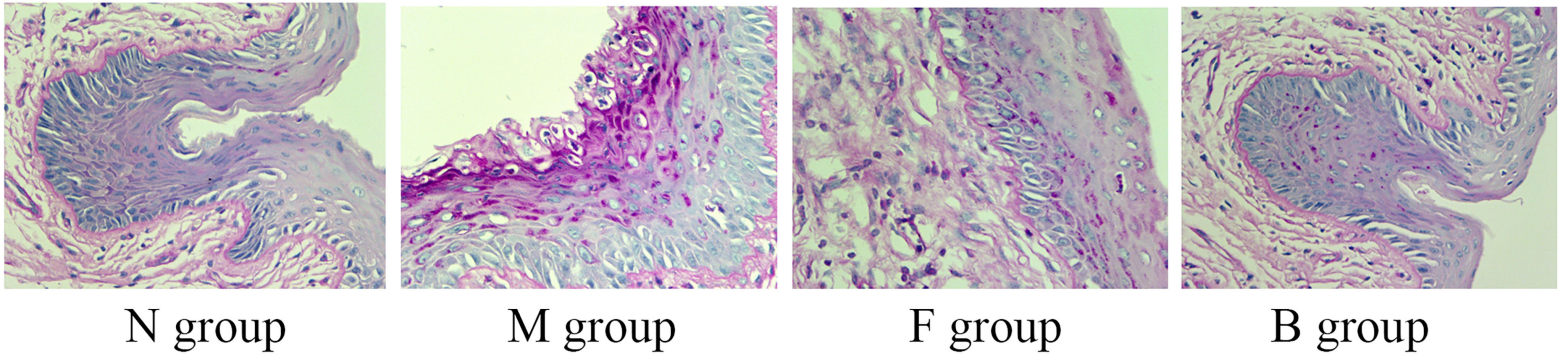
Representative histological micrographs of PAS staining of vaginal tissue in groups N, M, F, and B on the seventh day after treatment.

Hyphae or spores appeared purple, and the nuclei appeared blue after PAS staining. After the seventh day of treatment, no hyphae were seen in group N, a large amount of hyphal adhesion was seen in group M, and there were significantly fewer adhesion hyphae or spores in groups B and F than in group M. There was no significant difference in hyphal adhesion between groups B and F, suggesting that boric acid gel and fluconazole inhibited hyphal reproduction (**Figure 6**).

## Discussion

CA is the primary causative agent of VVC. CA typically colonizes healthy people’s mouth, gastrointestinal tract, and vaginal mucosa; however, it may cause symptomatic *Candida* vaginitis in immunocompromised hosts (18). In the vaginal mucosa, there is a balance between fungal virulence and host immunity that requires a coordinated movement of innate and acquired immune systems (19, 20). Cell-acquired immunity, represented by Th lymphocytes, plays a vital role in regulating CA infection (21).

In recent years, the diagnosis rate of clinical VVC patients continues to increase, but due to the lack of understanding of the pathogenesis of VVC and the existence of problems such as drug resistance in clinical treatment, the accurate selection of antifungal drugs has brought some troubles, and the corresponding economic burden for patients and the society. Thus, the present study was designed to focus on the immune mechanism of VVC caused by *Candida albicans* infection and the therapeutic effect and mechanism of boric acid gel.

The construction of VVC model is the key to the success of this study. There have been many research reports on the establishment of VVC animal models at home and abroad, and the animals mainly mentioned are rats, mice, rabbits, monkeys and so on. The advantages of rat as an animal model lie in its large volume and easy operation and sampling. However, rat ovaries have an impact on the pseudoestrus induced by exogenous estrogen, and both ovaries need to be removed before injection of exogenous estrogen, so the operation is slightly complicated (22). The characteristics of rabbits and rats are similar. At the same time, the economic cost of rabbits is high, so the adoption of rabbits is less. Monkey model construction does not require the creation of false estrus, but as a primate, its feeding conditions are insufficient, and its price is expensive, and its application is rare (23). In the establishment of VVC animal model in mice, the influence of ovaries on the establishment of pseudoestrus can be ignored, and the intervention of exogenous estrogen can only be given (1). Moreover, the operation steps are few and the materials are economical. Mice are also used in most basic experiments, and the data obtained in experiments are highly reliable. The experiment, using the method of Zhang X, et al. (17) for reference, mice VVC infection model was successfully constructed, which showed the presence of vulval-hyperemia, swelling, increased exudation, erosion or ulcers. Fungal microscopic examination and culture of vaginal lavage fluid in mice indicated successful modeling, which laid a good foundation for further study of the experiment.

Currently, it is generally believed that the pathogenesis of VVC is not only related to the virulence of the strain, but also closely related to the immune response of patients. Therefore, it is necessary to study the pathogenesis and progression of immunity and VVC. Studies have found that the host innate immune system can gradually mobilize local adaptive immune cells CD4+T helper cells (Th) and their secreted cytokines to play a role (4). Th cells can be divided into Th1, Th2, Th17 and other subtypes according to the differences of secreted cytokines.Th1-type immune cells secrete pro-inflammatory cytokines such as IL-2, IL-12, IL-23, IFN-γ, and TNF-α. Of these, the signature factors are IL-12 and IFN-γ. IL-12 is primarily involved in B cell-mediated immune responses to exert antibacterial effects. IFN-γ is produced by T lymphocytes and natural killer cells, inducing innate immune cells to improve resistance to CA (24, 25). IFN-γ also exerts anti-inflammatory effects by modulating the balance between Th1/Th2 cells (i.e., by activating Th1 cell activation and proliferation and inhibiting Th2 cell action) (6, 26). Th17-type immune cells secrete various cytokines to participate in antibacterial reactions; these include IL-17A/F, IL-6, and IL-22; of these, the typical representative is IL-17. IL-17 induces innate immune cells to participate in the inflammatory responses by promoting macrophages and epithelial cells to produce chemokines with recruitment effects and increase overall anti-infectivity (6, 17). Several regulators are required to differentiate Th17 cells, including TGF-β, IL-6, and IL-21 (27). IL-4 and IL-10, as representative factors of Th2 adaptive immunity, play an important role in candida infection. Il-4 can play a role in the elimination of pathogenic fungi, especially in the early stage (28–30). And also partially inhibits the activation of IFN-γ on macrophages (31), further weakening the uptake capacity of innate immune cells to strains. In addition, IL-10 can also inhibit the differentiation of Th0 cells into Th1 cells and their exclusive cytokines secretion and proliferation (29).

The most interesting finding was that cytokines-dependent Th1, Th2 and Th17-types responses were strongly correlated with the occurrence and development of VVC. Group M presented that expression levels of IFN-γ, IL-17, IL-6, TGF-β, IL-4, and IL-10 were significantly elevated in vaginal secretions after CA vaginal infection. With the prolongation of infection time, the expression of all cytokines remained high. A possible explanation for this might be that Th1, Th2, and Th17 cytokines played an antifungal role in the early stage, while the continued high expression of Th2 cytokines may antagonized the Th1/Th17 response and weakened the local antifungal effect in the later stage. Meanwhile, Th2 cytokines may be involved in the maintenance of infection and reduce the clearance rate of pathogenic fungi. These relationships may partly be further explained by subsequent knockout and transcription of representative Th cytokines related genes.

Boric acid, as a fungal bacteriostatic agent, has been used clinically in the treatment of vulvovaginal and ear canal infections. Some studies have shown that boric acid has good efficacy in the treatment of VVC, is a safe and economical choice, and is basically a first-line alternative to azole drug resistance (13, 14). In the present study, we established a VVC infection model and found that redness and exudation were observed at the local modeling site of mice, and PAS staining of mouse vaginal tissue was found significant local fungal load after CA infection. After treatment with boric acid gel and fluconazole gel, the symptoms of vulvovaginal redness and erosion were alleviated, the colony culture counts in the lavage solution were significantly reduced, the content of mycelia and spores in PAS staining group was significantly reduced compared with that in M group. Suggesting that the local effects of boric acid treatment was greater than fluconazole.

Azole is the most commonly used treatment for VVC in clinic. Representative drug fluconazole is a triazole traditional antifungal drug, which can play an antifungal effect by increasing cell membrane permeability and regulating host immune response. Studies have proved that fluconazole can promote the Th1 response of the host, inhibit the secretion of Th2 cytokines, and regulate the balance and coordination of Th1/Th2 antifungal effects (15, 32). Compared with group M, expression levels of cytokines IFN-γ, IL-17, IL-6, and TGF-β were significantly increased after pharmacological intervention, and expression levels of the anti-inflammatory factors IL-4 and IL-10 were significantly reduced. These results indicate that either boric acid gel or fluconazole gel may exhibit immunomodulatory functions on antifungal Th cell responses, expression levels of proinflammatory factor were significantly elevated and anti-inflammatory cytokine were inhibited in vaginal secretions after medication, suggested the shift of immune balance was modulated toward Th1 and Th17, thereby eliminating local *C. albicans*.

Although it is clear from the present study that boric acid gel can act in synergism to inhibit and Th2 responses a progressive infection, and promote Th1 and Th17 responses, the mechanism underlying this effect remains undefined. Given the detrimental role of IL-4 and IL-10 in murine candidiasis, inhibition of cytokines of Th2-type function during infection could be beneficial. It is possible that the reduction of the fungus burden that occurs as a result of antifungal therapy may further decrease the production of IL-4 and IL-10 in infected mice, as already demonstrated, thus amplifying the effect of boric acid gel therapy. The relative absence of inhibitory cytokines, such as IL-4 and IL-10, may allow for the expansion of Th1,Th17 cytokines.

Our study reveals a new immunomodulatory function for boric acid gel: the ability to potentiate antifungal effect by regulating cytokines of Th dependent. This effect is obtained, these findings further indicate that interfering with the balance of Th cell subsets and their cytokines may alter the outcome of systemic and mucosal candidiasis. Results from a preliminary experiment indicate that the immunomodulatory function of boric acid gel as well as fluconazole gel were retained in mice infected with *C. albicans*, and shifted the focus of local immune response balance to Th1 and Th17. If boric acid gel maintains its immunomodulatory properties, it can provide a reliable scientific basis for clinical treatment of mucosal fungal infection.

## Data Availability Statement

The raw data supporting the conclusions of this article will be made available by the authors, without undue reservation.

## Ethics Statement

The animal study was reviewed and approved by Biomedical Ethics Committee of Medical School of Hebei University of Engineering.

## Author Contributions

XG and TJ contributed to conception and design of the study. XL and ZL revised the manuscript. XG wrote the first draft of the manuscript. TJ, XG, and YX wrote sections of the manuscript. YC and YX organized the database. YL and HG performed the statistical analysis. All authors contributed to manuscript revision, read, and approved the submitted version.

## Conflict of Interest

The authors declare that the research was conducted in the absence of any commercial or financial relationships that could be construed as a potential conflict of interest.

## Publisher’s Note

All claims expressed in this article are solely those of the authors and do not necessarily represent those of their affiliated organizations, or those of the publisher, the editors and the reviewers. Any product that may be evaluated in this article, or claim that may be made by its manufacturer, is not guaranteed or endorsed by the publisher.

## References

[1] Willems HME Ahmed SS Liu J Xu Z Peters BM. Vulvovaginal Candidiasis: A Current Understanding and Burning Questions. J Fungi (Basel) (2020) 6(1):27. doi: 10.3390/jof6010027 PMC7151053 32106438

[2] Martin-Cruz L Sevilla-Ortega C Benito-Villalvilla C Diez-Rivero CM Sanchez-Ramón S Subiza JL. A Combination of Polybacterial MV140 and *Candida Albicans* V132 as a Potential Novel Trained Immunity-Based Vaccine for Genitourinary Tract Infections. Front Immunol (2021) 11:612269. doi: 10.3389/fimmu.2020.612269 33552074 PMC7858650

[3] De Gregorio PR Parolin C Abruzzo A Luppi B Protti M Mercolini L. Biosurfactant From Vaginal Lactobacillus Crispatus BC1 as a Promising Agent to Interfere With *Candida* Adhesion. Microb Cell Fact (2020) 19(1):133. doi: 10.1186/s12934-020-01390-5 32552788 PMC7302142

[4] Camilli G Blagojevic M Naglik JR Richardson JP. Programmed Cell Death: Central Player in Fungal Infections. Trends Cell Biol (2020) 31(3):179–96. doi: 10.1016/j.tcb.2020.11.005 PMC7880884 33293167

[5] Ardizzoni A Sala A Colombari B Giva LB Cermelli C Peppoloni S. Perinuclear Anti-Neutrophil Cytoplasmic Antibodies (pANCA) Impair Neutrophil Candidacidal Activity and Are Increased in the Cellular Fraction of Vaginal Samples From Women With Vulvovaginal Candidiasis. J Fungi (Basel) (2020) 6(4):225. doi: 10.3390/jof6040225 PMC7712103 33081210

[6] Ochs HD Oukka M Torgerson TR. TH17 Cells and Regulatory T Cells in Primary Immunodeficiency Diseases. J Allergy Clin Immunol (2009) 123(5):977–983; quiz 984-985. doi: 10.1016/j.jaci.2009.03.030 19410687 PMC2708116

[7] Logiodice F Lombardelli L Kullolli O Haller H Maggi E Rukavina D. Decidual Interleukin-22-Producing CD4+ T Cells (Th17/Th0/IL-22+ and Th17/Th2/IL-22+, Th2/IL-22+, Th0/IL-22+), Which Also Produce IL-4, Are Involved in the Success of Pregnancy. Int J Mol Sci (2019) 20(2):428. doi: 10.3390/ijms20020428 PMC6359245 30669479

[8] Khosravi AR Shokri H Darvishi S. Altered Immune Responses in Patients With Chronic Mucocutaneous Candidiasis. J Mycol Med (2014) 24(2):135–40. doi: 10.1016/j.mycmed.2014.01.062 24582143

[9] Nguyen TNY Matangkasombut O Ritprajak P. Differential Dendritic Cell Responses to Cell Wall Mannan of Candida Albicans, Candida Parapsilosis, and *Candida Dubliniensis* . J Oral Sci (2018) 60(4):557–66. doi: 10.2334/josnusd.17-0426 30429436

[10] Chen X Li T Wang FJ Shang CG Zhang X Bai HH. [Changes of Local Vaginal Immune Regulation in Rats Infected With Vulvovaginal Candidiasis]. Zhonghua Fu Chan Ke Za Zhi (2019) 54(5):330–7. doi: 10.3760/cma.j.issn.0529-567x.2019.05.008 31154715

[11] Powell A Ghanem KG Rogers L Zinalabedini A Brotman RM Zenilman J. Clinicians' Use of Intravaginal Boric Acid Maintenance Therapy for Recurrent Vulvovaginal Candidiasis and Bacterial Vaginosis. Sex Transm Dis (2019) 46(12):810–2. doi: 10.1097/OLQ.0000000000001063 PMC6878170 31663976

[12] Marrazzo JM Dombrowski JC Wierzbicki MR Perlowski C Pontius A Dithmer D. Safety and Efficacy of a Novel Vaginal Anti-Infective, TOL-463, in the Treatment of Bacterial Vaginosis and Vulvovaginal Candidiasis: A Randomized, Single-Blind, Phase 2, Controlled Trial. Clin Infect Dis (2019) 68(5):803–9. doi: 10.1093/cid/ciy554 PMC6376090 30184181

[13] Khameneie KM Arianpour N Roozegar R Aklamli M Amiri MM. Fluconazole and boric acid for treatment of vaginal candidiasis–new words about old issue. East Afr Med J (2013) 90(4):117–23. doi: 10.1016/j.mycmed.2014.01.062 26866095

[14] Guaschino S De Seta F Sartore A Ricci G De Santo D Piccoli M. Efficacy of Maintenance Therapy With Topical Boric Acid in Comparison With Oral Itraconazole in the Treatment of Recurrent Vulvovaginal Candidiasis. Am J Obstet Gynecol (2001) 184(4):598–602. doi: 10.1067/mob.2001.111938 11262459

[15] Cenci E Mencacci A Del Sero G Bistoni F Romani L. Induction of Protective Th1 Responses to *Candida Albicans* by Antifungal Therapy Alone or in Combination With an Interleukin-4 Antagonist. J Infect Dis (1997) 176(1):217–26. doi: 10.1086/514027 9207370

[16] Baoyuan LI Jiang H Bian R Wang B Sun S Gao X. Preparation and Quality Control of Compound Fluconazole Gel. China Pharmacist (2016) 19(9):1682–5. doi: 10.1093/cid/ciy554.

[17] Zhang X Li T Chen X Wang S Liu Z. Nystatin Enhances the Immune Response Against *Candida albicans* and Protects the Ultrastructure of the Vaginal Epithelium in a Rat Model of Vulvovaginal Candidiasis. BMC Microbiol (2018) 18(1):166. doi: 10.1186/s12866-018-1316-3 30359236 PMC6202846

[18] Moyes DL Richardson JP Naglik JR. *Candida albicans*-Epithelial Interactions and Pathogenicity Mechanisms: Scratching the Surface. Virulence (2015) 6(4):338–46. doi: 10.1080/21505594.2015.1012981 PMC4601190 25714110

[19] Drell T Lillsaar T Tummeleht L Simm J Aaspõllu A Väin E. Characterization of the Vaginal Micro- and Mycobiome in Asymptomatic Reproductive-Age Estonian Women. PLoS One (2013) 8(1):e54379. doi: 10.1371/journal.pone.0054379 23372716 PMC3553157

[20] Höfs S Mogavero S Hube B. Interaction of *Candida albicans* With Host Cells: Virulence Factors, Host Defense, Escape Strategies, and the Microbiota. J Microbiol (2016) 54(3):149–69. doi: 10.1007/s12275-016-5514-0 26920876

[21] Netea MG van der Meer JW Sutmuller RP Adema GJ Kullberg BJ. From the Th1/Th2 Paradigm Towards a Toll-Like Receptor/T-Helper Bias. Antimicrob Agents Chemother (2005) 49(10):3991–6. doi: 10.1128/AAC.49.10.3991-3996.2005 PMC1251502 16189071

[22] Fidel PL Jr Cutright JL Tait L Sobel JD. A Murine Model of Candida Glabrata Vaginitis. J Infect Dis (1996) 173(2):425–31. doi: 10.1093/infdis/173.2.425 8568305

[23] Wenjie T. Establishment of a Mouse Model of Recurrent Vulvovaginalcandidiasis and the Study of the Changes of Vaginal Localimmune Factors in the Different Stages. Yunnan: Kunming Medical University (2020). doi: 10.27202/d.cnki.gkmyc.2020.000759

[24] Guarda G Braun M Staehli F Tardivel A Mattmann C Förster I. Type I Interferon Inhibits Interleukin-1 Production and Inflammasome Activation. Immunity (2011) 34(2):213–23. doi: 10.1016/j.immuni.2011.02.006 21349431

[25] Shi D Li D Wang Q Kong X Mei H Shen Y. Silencing SOCS1 in Dendritic Cells Promote Survival of Mice With Systemic *Candida Albicans* Infection *via* Inducing Th1-Cell Differentiation. Immunol Lett (2018) 197:53–62. doi: 10.1016/j.imlet.2018.03.009 29581081

[26] Li T Niu X Zhang X Wang S Liu Z. Baofukang Suppository Promotes the Repair of Vaginal Epithelial Cells in Response to *Candida albicans* . AMB Express (2016) 6(1):109. doi: 10.1186/s13568-016-0281-1 27830496 PMC5102987

[27] Korn T Bettelli E Oukka M Kuchroo VK. IL-17 and Th17 Cells. Annu Rev Immunol (2009) 27:485–517. doi: 10.1146/annurev.immunol.021908.132710 19132915

[28] Noben-Trauth N Hu-Li J Paul WE. IL-4 Secreted From Individual Naive CD4+ T Cells Acts in an Autocrine Manner to Induce Th2 Differentiation. Eur J Immunol (2002) 32(5):1428–33. doi: 10.1002/1521-4141(200205)32:5<1428::AID-IMMU1428>3.0.CO;2-0 11981831

[29] Elahi S Pang G Clancy R Ashman RB. Cellular and Cytokine Correlates of Mucosal Protection in Murine Model of Oral Candidiasis. Infect Immun (2000) 68(10):5771–7. doi: 10.1128/IAI.68.10.5771-5777.2000 PMC101536 10992484

[30] Mencacci A Spaccapelo R Del Sero G Enssle KH Cassone A Bistoni F. CD4+ T-Helper-Cell Responses in Mice With Low-Level *Candida Albicans* Infection. Infect Immun (1996) 64(12):4907–14. doi: 10.1128/iai.64.12.4907-4914.1996 PMC174467 8945525

[31] Stevenhagen A van Furth R. Interferon-Gamma Activates the Oxidative Killing of *Candida Albicans* by Human Granulocytes. Clin Exp Immunol (1993) 91(1):170–5. doi: 10.1111/j.1365-2249.1993.tb03374.x PMC1554646 8419079

[32] Ren X Liu W Liu Y. Effects of Fluconazole on the Clinical Outcome and Immune Response in Fungal Co-Infected Tuberculosis Patients. Microb Pathog (2018) 117:148–52. doi: 10.1016/j.micpath.2018.02.015 29432913

